# Binary titanium alloys as dental implant materials—a review

**DOI:** 10.1093/rb/rbx027

**Published:** 2017-09-23

**Authors:** Xiaotian Liu, Shuyang Chen, James K.H. Tsoi, Jukka Pekka Matinlinna

**Affiliations:** 1 Department of Orthodontics, Tianjin Stomatological Hospital of Nankai University, Tianjin, P. R. China; 2 Dental Materials Science, Faculty of Dentistry, The University of Hong Kong, Prince Philip Dental Hospital, 34 Hospital Road, Hong Kong SAR, P. R. China; 3 Department of Prosthodontics, Tianjin Stomatological Hospital of Nankai University, Tianjin, P. R. China

**Keywords:** **:** binary Ti, Ti alloy, dental implant, biocompatibility

## Abstract

Titanium (Ti) has been used for long in dentistry and medicine for implant purpose. During the years, not only the commercially pure Ti but also some alloys such as binary and tertiary Ti alloys were used. The aim of this review is to describe and compare the current literature on binary Ti alloys, including Ti–Zr, Ti–In, Ti–Ag, Ti–Cu, Ti–Au, Ti–Pd, Ti–Nb, Ti–Mn, Ti–Mo, Ti–Cr, Ti–Co, Ti–Sn, Ti–Ge and Ti–Ga, in particular to mechanical, chemical and biological parameters related to implant application. Literature was searched using the PubMed and Web of Science databases, as well as google without limiting the year, but with principle key terms such as ‘ Ti alloy’, ‘binary Ti ’, ‘Ti-X’ (with X is the alloy element), ‘dental implant’ and ‘medical implant’. Only laboratory studies that intentionally for implant or biomedical applications were included. According to available literatures, we might conclude that most of the binary Ti alloys with alloying <20% elements of Zr, In, Ag, Cu, Au, Pd, Nb, Mn, Cr, Mo, Sn and Co have high potential as implant materials, due to good mechanical performance without compromising the biocompatibility and biological behaviour compare to cp-Ti.

## Introduction

Titanium (Ti) is a transition metal and element with the atomic number of 22. Ti has a lustrous finishing and characterized with silver colour, low density and high strength. It has a high ability to resist corrosion in various media such as sea water, aqua regia and chlorine [1]. Ti is also claimed to be biocompatible since it is non-toxic nor rejected by the human body. Thus, Ti and its alloys can be used in various medical usages, e.g. surgical implements and implants, and in dentistry, e.g. abutment, prostheses and orthodontic wires [2]. In particular, study [3] has shown Ti-made hip balls and sockets (as a joint replacement) could stay in patients’ body for more than 20 years. Furthermore, Ti has the inherent ability to osseointegrate, which enable Ti to be used for orthopaedic implant applications [4]. In addition, Ti has a low modulus of elasticity (i.e. Young’s modulus) which matched closely to the bone. As a result, loads from skeletal could be more evenly distributed between bone and implant, and led to a lower incidence of bone degradation which is due to [1] stress shielding and [2] periprosthetic bone fractures happened at the orthopaedic implants boundaries [5]. Despite the stiffness of Ti is more than twice that of bone, which might consequently deteriorate the adjacent bone due to a reduced load was asserted on the bone [6], Ti still deemed to be a material that is being used in medicine.

Ti is a dimorphic metal with two phase, α and β phase. α-Ti is hexagonal close-packed (hcp) crystal lattice, and β-Ti is body-centered cubic (bcc) lattice. During the processing, Ti exists as α-Ti when the temperature is lower than 883 °C. When Ti is heated exceeding 883 °C, the atoms in the hcp crystal lattice packed closer with each other and become β-Ti [7–9]. α-Ti is strong, high strength at high temperatures (<883 °C) and has a good weldability. However, it is difficult to work and heat treatable. The tensile strength is about 330–860 MPa and fracture toughness >70 MPa m^−^^1/2^. For β-Ti, beta phase was observed at room temperature after quenching, or sometimes even upon air cooling. Thus, it is ready for cold working (forming), and could be solution-treated, quenched and aged to give higher strength together with low ductility. However, its fatigue performance is poor. The tensile strength is about 1220–1450 MPa and fracture toughness of >50 MPa m^−^^1/2^.

**Figure 1.**
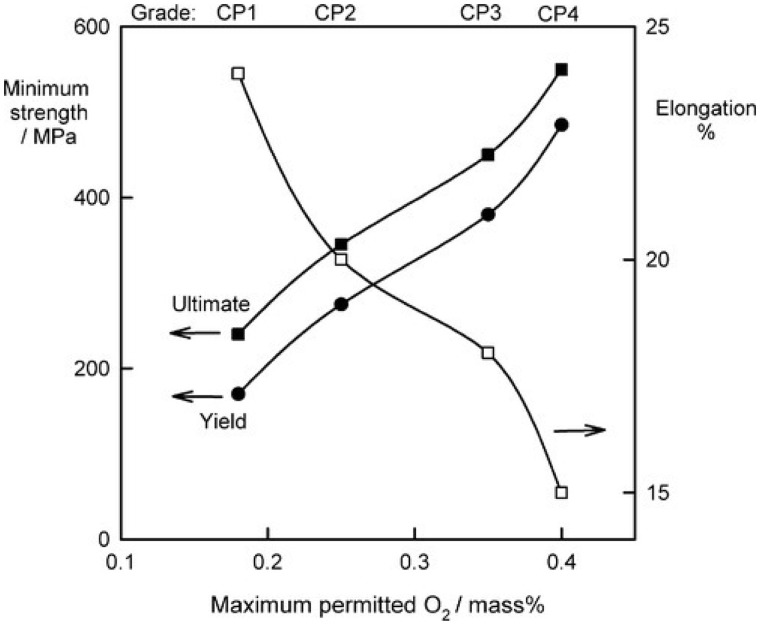
the strength and oxygen contents variation on cp-Ti, adapted from [9]

Indeed, the commercially pure Ti (cp-Ti) is divided into four Grades from 1 to 4, according to the purity and the processing oxygen content [10]. These different grades of cp-Ti have various corrosion resistance ability, ductility, and strength (Fig. 1) . For example, Grade 1 cp-Ti, which is processed with the least oxygen content (around 0.18%), has the highest purity, the best corrosion resistance ability and formability. However, the overall mechanical strength is the lowest. On the other hand, the Grade 4 cp-Ti, which is processed with the most oxygen content (around 0.4%), has the highest strength and moderate formability. Due to the highest exhibited strength, thus most Ti implants are made from Grade 4 cp-Ti.

## Ti as an implant material

In order to replace a missing tooth, a lot of materials, such as cobalt–chromium (Co–Cr, Vitallium) and stainless steel, had been attempted to make an implant. The development of materials science and technology improved the materials for implant application. Nowadays, Ti becomes the most popular implant material due to its advantages.

In fact, Ti is widely and successfully used as an implant material primarily due to various factors. Ti is biologically inert, able to bond with osteoblasts and has excellent biocompatibility. The spontaneously formed oxide layer, i.e. Ti oxides (TiO_x_) as film, is very stable and could separate the bulk Ti material from its surrounding. Thus, Ti has a high ability to resist the corrosion. The TiO_x_ layer is typically around 3–10 nm thick that stably stayed onto the Ti surface, and the oxide film on the surface [11] can absorb calcium and phosphate ions and induce some protein to form apatite, i.e. promotion of osseointegration. However, this oxide film layer is very thin and easily to be destroyed. Thus, various attempts have been done to protect TiO_x_ coating. For example, some artificial methods, such as electrochemical oxidation [12], anodic oxidation [13] and heating under atmospheric pressure [14] or *in vacuo* [15] are proposed to thicken the oxide layer (but still <10 nm) which could also prevent Ti ions leakage that cause the protein denaturation and necrosis of tissue cells [16].

Despite the thickness of TiO_2_ (<10 nm) could be controlled uniformly, fundamentally, at the atmospheric environment, small portion of Ti and Ti-OH at the surface would chemically react (by chemisorption) with the water moisture which might lead weakly bounded physisorbed water on the surface. In the condition of multivalent Ti (e.g. Ti^4+^) metal, together with the physisorbed water that proceed with the equilibrium become hydroxide (OH^−^) and hydronium (H^+^) ions, Ti is readily to form Ti-OH:

(1) $$H_{2}O\;↔{\;H}^{+}+{\;\text{OH}}^{-}$$

(2) $${\text{Ti}}^{4+}+{\;4\text{OH}}^{-}↔\;4\text{Ti}-\text{OH}$$

Then, the Ti-OH is likely to undergo further hydrolysis [17]:

Ti-OH + H_2_O ↔ [Ti-O]^−^ + H_3_O^+^

(3) $$\text{Ti}-\text{OH}+{\;H}_{2}O\;↔\;{\left[\text{Ti}-O\right]}^{-}+{\;H}_{3}O^{+}$$

(4) $$\text{Ti}-\text{OH}+{\;H}_{2}O\;↔\;{\left[\text{Ti}-{\text{OH}}_{2}\right]}^{+}+{\;\text{OH}}^{-}$$

In theory, equilibrium reaction Equation (3) would lead to the formation of basic type of Ti oxide ([Ti-O]^−^) which incur negative charge on the surface, and the acidic type [Ti-OH_2_]^+^ incur positive charge for Equation (4) [18]. Studies [19–21] had shown that the isoelectric point (IEP) for these Ti oxide at surface ranged from 5.0 to 6.7. The equilibrium reactions Equations (3) and (4) suggested that, under the acidic pH lower than IEP, the predominant oxide specie would be [Ti-OH_2_]^+^. Thus, surface treatment methods such as the acid etching could not only roughen the surface that provide a harbouring site for negatively charged osteoblast [22], but also induce the formation of hydroxylated Ti oxide form [Ti-OH_2_]^+^, which is hydrophilic and documented for biological activity enhancement [23]. Thus, keeping a positive acidic [Ti-OH_2_]^+^ on Ti surface could be a good strategy for Ti-osteoblast bonding without the addition of bi-positively charged growth factors/proteins [24].

However, during the storage and the equilibrium nature of the oxide, the nano-oxide layer will become thicker due to the time and quality of exposed storage atmosphere [17]. In such a case the hydroxylated oxide would be diluted and deprotonated, i.e. more [Ti-O]^−^ is formed, and thus hydrophilicity and nanostructure would be affected. Therefore, in order to maintain the level of [Ti-OH_2_]^+^, a storage of acidic medium is recommended which might be a tactic in the commercial product ‘SLActive’ (Straumann, Basel, Switzerland) that use an acidified saline (i.e. 0.9% NaCl, pH 4–6) to preserve effectively the hydrophilicity and nanostructure [25].

In general, titanium is a good choice for intraosseous applications not only due to the biocompatibility, but also mechanically titanium could be processed and machined in a rapid manner such that the shapes and sizes could be easily controlled. Nevertheless, one of the disadvantages of the titanium could be the aesthetic problem since Ti is grey in colour, such that the dark colour would be seen through the thin mucosa if the soft tissue situation is not optimal. Ti also proceeded with some other drawbacks, e.g. low deformability and wear resistance, and high reactivity with the surrounding impurities (such as oxygen and nitrogen) at elevated temperatures [26, 27]. Moreover, Ti is proven to release Ti ions under the physiological condition, such that the presence of citrate and lactate would increase the Ti levels, as well as assisting the binding between Ti and transferrin [28]. Another study [29] showed that the speciated compound formed between Ti, citrate and transferrin was stable and non-toxic. However, this compound is transportable within the body via blood stream, decreases the pH of endosome, and weakens the Ti implant integrity. All these effects in long term are not known. Thus, cautious should be taken when using Ti in the implant application.

One of the tactics—alloying Ti with a variety of elements—might become viable to enhance some of these properties, such as increase the corrosion resistance, lower the modulus of elasticity, and improve the machinability. This is because the Ti-alloys properties are related to their respective phases/crystalline structures, such that by adding some alloying elements could stabilize certain phases. In addition, some metal substrates could combine with this oxide layer in order to prevent absorption and disintegration of coating [30, 31]. Thus, alloying the titanium might be a strategy to improve the mechanical and other properties [10].

## Binary titanium alloys

Commonly, alloy could broadly defined as ‘a mixture of a metal and another element(s) which has metallic bonding character’. In titanium, a lot of attempts, such as using Silver (Ag), Aluminium (Al), Copper (Cu), Iron (Fe), Vanadium (V) and Zinc (Zn), have been done in order to improve the material.

In fact, as aforementioned, the titanium has three forms: α, β and α-β. With the addition of the alloying elements, the phase compositions could be adjusted and thus change the bulk Ti-alloy properties. For example, Al acts as α-phase stabilizers which could improve the strength and lower the weight of the alloy. V is β-phase stabilizer that could improve the ductility and formability. Thus, by adding of the Al and V, the temperature for α-phase transform to β-phase has also been changed to a range, i.e. both α and β phases exist in the temperature range. The most commonly used Ti-alloy in dental implant [7, 32] is Ti–6Al–4V, which is also known as Grade V titanium alloy, composed of 6 and 4% of aluminium and vanadium, respectively, together with addition of maximum 0.25% of iron and 0.2% of oxygen. The remaining of the alloy is titanium. When compared with cp-Ti, Ti–6Al–4V has an excellent yield strength and fatigue properties, excellent corrosion resistance ability and lower elastic modulus.

Ti–6Al–4V (commonly also known as Grade V Titanium) is one of the most commonly used tertiary titanium alloy, that is usable as a biomedical implant. As the symbol implies, Ti–6Al-4V has the composition of 6 wt% aluminium and 4 wt% vanadium. In particular, Ti–6Al–4V alloy has a higher strength that could be used in various applications, such as for anchorage stems of femoral components, together with Co–Cr–Mo alloy or Al_2_O_3_ ceramic ball heads. However, Ti-6Al–4 V alloy has the disadvantage of low wear resistance [33], high elastic modulus [34] (still approximate 4–10 times of human bone) and low shear strength [35] that could impair the usage as implant and as in screw form. Such a phenomenon is termed as ‘stress shielding effect’ [36], which is a due to the stiffness mismatch between implant material and surrounding bone. Long-term studies [37, 38] had shown that when load transfer from artificial implant is insufficient to adjacent remodeling bone, bone resorption might happen and eventually the prosthetic device is loosened. Therefore, suitable surface treatments [33–35] were deemed to be necessary to improve the situation.

Despite Ti–6Al–4V alloy has been widespread in use as an implant biomaterial, study [39] has shown the alloy could release of aluminium and vanadium ions [40]. In particular, vanadium exhibits a high cytotoxicity [41] and aluminum may even induce senile dementia [41]. This said, these leachable metal ions might cause various health issues such as allergic, cytotoxic effect and even neurological disorders. As implant is not installed in body for a short time, some health problems, such as Alzheimer diseases, osteomalacia and peripheral neuropathy should not be under-estimated and the use of alloys for implant application should be cautiously picked. Extra attention should be paid when alloying with the Titanium.

Therefore, to successfully use the new materials in dentistry, a continuous development of new Ti-based alloys with ideal properties, e.g. without any toxic effects, is desirable. Thus, some other alloys, particularly binary Ti-alloys such as Ti–Nb [42–44], Ti–Ag [45, 46], Ti–Au [47], Ti–Mn [48], Ti–Cr [49, 50], Ti–Mo [51], Ti–Sn [52], Ti–Zr [53–55], Ti–Co [56], Ti–Pd [57] and Ti–Cu [58] have been developed. The processing technique and chemical composition of these Ti-alloys would influence their microstructure, and hence the mechanical properties. In the following sections, several binary Ti-alloys are briefly introduced, discussed and described.

### Ti–Zr

Zirconium (Zr), is a neutral element when dissolved in Ti. Zirconium belongs to Group 4 (according to new IUPAC name) in the periodic table, which is the same as titanium and hafnium, have similar chemical structure and properties. Thus, they have been recognized as non-toxic and non-allergic. Zirconium is a transition metal with atomic number 40 and atomic weight of 91.22 amu. Being as a greyish-white lustrous metal, Zirconium has extremely high melting (1857 °C) and boiling (4409 °C) points. Zirconium has a great resistance to corrosion, which is similar to Titanium, and is therefore highly biocompatible [59] since the both metal surfaces form a stable oxide layer on their surface within nanoseconds when expose to oxygen. Thus, the oxidation passivates the materials. However, Zirconium could not be used in dentistry in its pure form.

Start from 1990s, the oxide form of zirconium, Zirconia (ZrO_2_), has started to use in dentistry, due to its biocompatibility and possesses the capacity to osseointegrate [60]. Zirconia is a ‘ceramic’ biomaterial which has been widespread in use as crown materials, fillers for resin composite and implant screw fixture. A very detailed and good review has been studied by Miyazaki *et al.* [61]. Despite in the 1970s the manufacturing process of zirconia had become completely controllable, for zirconia to be usable in dentistry, the materials should be manufactured following many different steps such as calcining zirconium compounds in order to exploit its high thermal stability [62]. However, the manufacturing process of dental zirconia is very strict and varies for each company. The final product may have different chemical compositions. Moreover, the companies have not provided intentionally extensive information about the materials and surface characteristics of the zirconia. Thus, difficulties always happen in the evaluation of the commercial products. For example, in Ho *et al.* [63] mentioned that China-made zirconia could not be fully sintered and might yield unexpectable bond strength result. Therefore, a careful selection of the zirconia is necessary.

An extensive review article [64] suggested that zirconium [sic] implants had an inferior degree of osseointegration than titanium analogue by using removal torque tests, and perhaps some surface modifications could restructure the implant that could allow the removal torque test values comparable with the titanium implants. Although the removal torque test values indeed highly depends solely on the surface structure (in terms of mechanical retention and biological interaction) than on the implant material itself [65], the atomic structural arrangement allows the torque performance in metal alloys better than ceramics. The reason for using zirconia implant is merely due to improvement on esthetic qualities in dental restorations. Thus, the development on Ti-alloys is still viable and active.

The binary phase diagram between titanium and zirconium presents a continuous solid solution. Indeed, the fusion temperature of the Ti (1670 °C) is lowered by increasing the amount of Zr (∼1640 and 1560 °C for 10 and 40 wt% Zr, respectively) regardless α-Ti or β-Ti. Thus, the casting process could be easily facilitated. In addition, from the knowledge of casting titanium [66], reducing the melting temperature of Ti could decrease its oxygenic reactivity and thus reduce the risk of inadequate filling of mould. Consequently, the temperature mismatch between the hot molten alloy and the much cooler investment materials might be less. Thus, less porosity could be developed.

Recently, due to their good corrosion resistance and biocompatibility, binary Ti–Zr alloys have been developed for dental applications [67–69] which claimed to be comparable with cp-Ti. Wen *et al.* [70] demonstrated that the Ti–Zr alloy has unique combinatorial properties of bioactivity, biocompatibility and mechanics that has a high potential in biomedical application. Furthermore, Ti–Zr alloy might significantly improve osteoblast adhesion [71], which is currently marketed as Roxolid (Straumann, Basel, Switzerland). Therefore, alloying titanium with zirconium deemed to be reasonable.

Kobayashi *et al.* [72] investigated some of the properties (hardness, tensile strength, and crystallinity via optical microscopy) of Ti-Zr binary alloys. Their results revealed that for at Ti-Zr with up to 50 wt% Zr, the hardness and tensile strength of all the alloys were higher than cp-Ti and pure zirconium. They indicated that Ti-Zr alloy could be useful in biomedical field as base alloy material. Indeed, Ho *et al.* [73] has claimed to develop an experimental Ti-10Zr alloy which has a higher hardness and better grindability than unalloyed titanium, but they then self-criticized the experimental Ti-Zr alloy has insufficient strength and elastic recovery (i.e. springback) properties for dental applications. Therefore, the processing of the Ti-Zr alloy could be a challenge.

### Ti–In

For a long time, Indium (In) has been used in Pd- and Ag-based porcelain-fused-metal (PFM) applications. In fact, during the firing process of porcelain, the indium oxides film would formed on the metal surface, which could be served as a ‘bonding agent’ between metal and porcelain [74, 75]. In addition, cytotoxicity tests revealed that dental alloys that contain indium are safe. Therefore, using indium as an alloying element to improve the alloy so as to improve the clinical performance of cp-Ti deemed to be reasonable.

Several studies on experimental Ti-In alloys has shown that Ti-In alloys were biocompatible. Further, with the addition of indium to Ti could improve clinical performance in terms of mechanical properties, corrosion resistance, and biocompatibility [76] of dental implant. According to a study with regards to a alkali-heat treated Ti–In–Nb–Ta alloy, the surface analysis revealed the alloy has a good bioactivity [77].

For binary Ti-In alloys, Wang [78] found that the passivation current densities in artificial saliva solutions for Ti–In alloys and cp–Ti exhibited the same order of magnitude. Furthermore, Ti–10In and Ti–15In (10 and 15 denote the respective Indium wt%) showed a transpassive behaviour and lower current densities at high potentials under the presence of NaF. Han *et al.* [79] has shown Ti-In alloys (5–20 wt% In) not only exhibit a similar corrosion resistance to cp-Ti by electrochemistry, but even a superior oxidation resistance compared to cp-Ti was revealed in Ti-In alloys. Therefore, Ti-In alloys might give a good or better corrosion resistance as cp-Ti.

Han *et al.* [79] has further studied the corrosion behaviour, mechanical properties and microstructures of Ti-In binary alloys, which confirm with another study by Wang *et al.* [78] that the strength and microhardness of the alloys were increased. Furthermore, in the experimental Ti–In alloys and cp-Ti in Wang [78] showed a good and similar cytocompatibility. Therefore, alloying of indium to titanium was effective fabricate a new alloy that might have a better mechanical properties without compromising its corrosion behaviour and cytocompatibility.

### Ti–Ag

Oh *et al.* [45] reported that Ti-Ag alloys had higher mechanical properties and corrosion resistance than Ti. They also reported that the toxicities of Ti–Ag alloy were similar to cp-Ti. Zhang *et al.* [46] confirmed this by using *in vitro* cytotoxicity test which showed Ti–Ag alloys and cp-Ti alloys seem to be cytocompatible with each other. They recommend that, for dental application, Ti–5Ag and Ti–20Ag might be more suitable according to the surface passive film and cytotoxicity viewpoints.

### Ti–Cu

Copper has been used for long time as dental casting alloys. Study [80] has shown the binary Ti–Cu alloy has a eutectoid structure at 7.0 mass%, such that in the titanium-rich region, an intermediate phase of Ti_2_Cu was found. Thus, Ti–Cu alloys that have a nearly eutectoid composition was expected to have higher strength and less ductility than cp-Ti.

Studies [80, 81] revealed that some Ti–Cu alloys might have superior mechanical properties than cp-Ti. In fact, this could be explained by various factors: (i) solid solution strengthens the titanium, and (ii) fine precipitation of intermetallic compounds, which might be similar to dental silver amalgam [6]! Despite study [80] revealed the experimental Ti–Cu alloys (up to 10.0% copper) increased both the yield and tensile strengths but decrease the ductility, the strength of Ti–20Ag and Ti–5Cu alloys was 1.6–2 times higher than that of cp-Ti.

Moreover, in terms of grindability, some Ti–Cu and Ti–Ag alloys had shown a better outcome than cp-Ti [47, 82]. At higher grinding speeds, some Ti-alloys with 5 and 10% Cu, and 20% Ag had been demonstrated significantly higher grindability, and even 2.6 times higher grinding rates than cp-Ti. Another report [83] has also demonstrated these titanium alloys has an excellent corrosion resistance similar to cp-Ti. Thus, the strength and the fabricability of these alloys might meet the requirements for partial dentures, clasps, and dental bridges.

### Ti–Au

Gold (Au), has been mainly used for dental prostheses in dentistry for a long time, particularly for casting, since gold has good corrosion resistance, suitable melting point, and could achieve appropriate mechanical properties by alloying [51]. Gold *per se* belongs to Group 11 in the periodic table, same as silver and copper. Gold, similar to silver and copper, stabilizes β-phase of titanium to a lower temperature according to the binary equilibrium phase diagrams.

According to the phase diagram, similar to the Ti-Cu and Ti-Ag alloys, the Ti-Au alloy has the eutectoid point with titanium. At the gold concentration of 15.3% the intermetallic compound Ti_3_Au forms. Therefore, gold, as an alloying element for titanium, might be expected to positively affect on the mechanical properties and grindability of titanium, similar to silver and copper.

Hwang *et al.* [84) found that Ti–10Au alloy showed the lowest value in galvanic corrosion current density test, i.e. very less likely to be corroded. Indeed, electrochemical techniques were utilized to study the galvanic corrosion effects of the passive surface layers on Ti–Au alloys. The impedance spectral results revealed the Ti–Au alloy passive layers consisted of the metal-defect, inner, intermediate and outer layers. The initial galvanic corrosion was driven by the outer layer of Ti–Au alloys. If a thin and porous superficial layer is formed, then the initial galvanic corrosion current density would be decreased, and vice versa. Thus, Ti–Au has the lowest galvanic corrosion current density meaning that only a thin and porous outermost layer was formed, and hence the least corrosion was found.

### Ti–Pd

Moser *et al.* [85] reported that Pd−Ti alloys have adequate corrosion resistance and mechanical property, e.g. hardness. Therefore, it could be utilized as a dental prosthetic alloy. Furthermore, Nakagawa *et al.* [86] utilized the anodic polarization curves and corrosion potentials experimental techniques to test Ti–Pd alloys (0.1–2 wt% of Pd) in artificial saliva containing 0.2% NaF, and reported that Ti-Pd alloys have a good corrosion resistance. Rosalbino *et al.* [87] utilized the impedance analysis method to test the electrochemical corrosion behaviour of some binary Ti alloys (*ca.* 1 at% Ag, Au, Pd and Pt) *vs.* commercial Ti-6Al-7Nb in the fluoridated artificial saliva. Takahashi *et al.* [81, 88] also reported the corrosion behaviour in 0.9 wt% NaCl and 1 wt% lactic acid solution by evaluating the microstructures of Ti–Ag and Ti–Au alloys using potentiodynamic polarization method. From all the above studies, it might be concluded that titanium alloyed with noble metal elements could all be sustained and survived in artificial corrosion. Thus, Ti-noble metal alloys might exhibit the potential advantages of: (i) biocompatibility, (ii) corrosion resistance and (iii) acceptable casting temperatures for PFM dental prostheses.

### Ti–Nb

Lee *et al.* [42] studied the corrosion behaviour, mechanical properties and microstructures of some binary Ti–Nb alloys (Nb up to 35 wt%) All Ti–Nb alloys had shown excellent resistance to corrosion ability. In another study by Kikuchi *et al.* [43], they examined the mechanical properties and grindability of Ti-Nb alloys dental cast. They found that the hardness, yield and tensile strengths of the Ti–Nb alloys (Nb > 10%) would significantly higher than that of cp-Ti, whilst the tensile strength was significantly lower. Only in Ti–30%Nb alloy, possibly due to precipitation, exhibited a significantly better grindability at low grinding speed with higher hardness, strength and Young’s modulus than cp-Ti.

### Ti–Mn

Manganese (Mn), is one of the trace elements that could be found in human body which has lower toxicity than Al and V. Mn can be added to tri-calcium phosphate by doping and become a bioceramic which showed a good cell compatibility [89]. In a recent study, plasma-spark sintered Ti-Mn alloys had demonstrated a promotion of the cell adhesion [90]. Similarly, utilizing the similar technique, Zhang *et al.* [48] has investigated the microstructures, mechanical properties and cytotoxicity of experimental Ti–Mn alloys compared with the cp-Ti and Mn metals. Accordingly, Ti–8Mn and Ti–12Mn alloys were prepared and they found that the doping of Mn in Ti could increase significantly the hardness and relative density of the Ti alloys, and also decrease the α to β phase transformation temperature. In addition, the Ti–8Mn alloy has demonstrated 86% cell viability comparable to that of the cp-Ti (93%). Thus, Mn could be a good alloying element for fabricating biomedical Ti alloy, such as to be used as bone substitutes and dental implants.

### Ti–Cr

Chromium (Cr), is well-known in dentistry due to the Co–Cr application that could control the anodic activity of the alloy, and could passivate Ti [49]. Takemoto *et al.* [91] reported, under the condition of saline solution with F^−^, the Ti–20Cr alloy had a greater corrosion resistant than cp-Ti. In addition, according to phase diagram, alloying high Cr content (46%) to Ti could reduce the liquidus temperature from the high melting point of cp-Ti (1670 °C) to a minimum (1410 °C), similar to Ti–Zr as previously discussed.

Hsu *et al.* [49] reported that the structure Cr content would affect greatly the Ti–Cr alloys. The cast cp-Ti has a hexagonal α phase. A metastable β phase will be retained with 5 wt% Cr, and equi-axed β phase would almost entirely be retained when Cr contents is higher than 10 wt%. Athermal ω phase was also start found from Ti–Cr alloy with >5 wt% of Cr. Largest quantity of ω phase, highest microhardness and best grindability were found in Ti–10Cr brittle alloy, since ω phase was found in the β matrix.

Ho *et al.* [50] investigated the casted Ti–Cr alloys with 5-30 wt% Cr. In particular, with respect to various amounts of Cr, these alloys behave obviously in great variety under deformation. For example, the Ti–20Cr alloy has similar bending strength with Ti–10Cr alloy which was about 1.8 times higher than cp-Ti. This might be due to the strengthening effect from the ω phase. Ho *et al.* also studied the fractography of the specimen, and fractographs of SEM showed that the Ti–10Cr alloy has: (i) coarse cleavage facets in the fractured surface; and (ii) some terrace-type morphology. In addition, the Ti–20Cr has shown to be ductile, such that it has even 460% more elastic recovery capability than cp-Ti, whereas other composition of Ti–Cr alloys did not exhibit such properties. As shown in their unetched optical micrographs, large number of slip bands was observed on the surfaces of the Ti–20Cr alloy. That said, slippage dislocations are the reasons of the Ti–20Cr alloy deformation. Therefore, if Ti–Cr alloys are needed to be use for prosthetic dental applications, many properties such as mechanical properties and deformation behaviour should be further studied.

### Ti–Mo

According to Bania [92] and Ho *et al.* [51], a minimum of 10 wt% of isomorphous β-stabilizing element is needed in order to stabilize β phase for a Ti-Mo alloy at room temperature. Below this percentage, the alloy consists of martensitic α″ phase that has a lower hardness than β-Ti-Mo. Ti–10Mo was tested to have the highest bending strength, and Ti–15Mo have the lowest modulus among the β-Ti-Mo [51], and even lower than other alloys such as Ti–6Al–4V, Ti–6Al–7Nb and 316 L stainless steel, and Grade IV cp-Ti [93], due to a fine grain bcc structure was obtained. Such an alloy was claimed to have a better processability [51]. Indeed, such a metastable β phase Ti–15Mo alloy, being manufactured by rapid quenching, has been marketed and sold for orthopaedic implant by Synthes USA. However, a careful selection about the concentration is necessary, since the existence of ω phase [94] at low concentration of Mo (<15%) might have low temperature ω → α transformation and thus affect the materials strength.

### Ti–Sn

Ti–Sn has been found nontoxic and nonallergic [95]. Thus, Tin (Sn) seems to be an alloying element that is safe to use with Ti. In addition, Sn could also strengthen Ti alloys [96], such that binary Ti–Sn alloys have been demonstrated some favorable mechanical properties that could be used as a metal for dental casting [52]. For example, experimental results indicated that all the 1–30 wt% Sn containing Ti–Sn alloys have a hcp α structure. Increasing the Sn concents could increase the Vickers hardness (H_V_) of the Ti–Sn alloys, e.g. 30 wt% Sn showed a high hardness value of 357 H_V_. To illustrate the CAD/CAM processability, grindability of the alloys was also studied [52]. In fact, the addition of Sn to cp-Ti would contribute the improvement of the grindability of Ti–Sn alloys such that a higher Sn concentration could be ground more readily, e.g. Ti–30Sn possessed 3.4 times higher grinding ratio than cp-Ti at grinding rate of 1200 m/min. However, the grindability of each metal or alloys was actually largely dependent on the grinding conditions. Thus, a careful interpretation of the experimental data is necessary.

### Ti–Co

Cobalt (Co), is a widely accepted biocompatible element [97, 98) that has been vastly used in dentistry such as Co–Cr base alloy. According to the phase diagram of Ti–Co, at its eutectic or near-eutectic composition, the melting range of the alloy could be significantly lowered. Thus, Ti–Co with low melting range [56] might have a good dental usability, particularly for base alloy which might have a good castability and less metal-mould reactions. Study [56] has demonstrated that, comparing to conventional dental casting alloys, experimental Ti–12Co alloy has similar mechanical properties as-cast, and a significant improvement in tensile strength was revealed after the post-casting heat treatment at >1000 °C, since the formation of brittle intermetallics was minimized. Therefore, Ti–Co would be a good candidate for the replacement of the base alloys with a better processability.

### Ti–Ge and Ti–Ga

Lin *et al.* [99] has studied casted Ti–Ge alloys from 2 to 20 wt% of Germanium, and concluded the 2 and 5 wt% has the highest potential for dental use, due to no toxicity, good mechanical performance, resist chemical corrosion and good processability. Similarly, from the same group of authors, Qiu *et al.* [100] has reported Ti–Ga alloys with 2 and 5 wt% Gallium showed a good potential for dental use due to the same reasons. It should be noticed that both studies did not disclose the processing temperature. According to the Ti–Ge phase diagram [101], for the presence of α-Ti the processing temperature should be <882 °C and >95.9 at% of Ti. The control for such a process is very technique sensitive, otherwise intermetallic compounds such as Ti_6_Ge_5_ and subsequently TiGe_2_ would be preferentially nucleated and crystalized [102]. For Ti–Ga, since gallium is a liquid metal with extremely low-melting point (29.77 °C), so the temperature for processing could be low. In fact, both studies revealed the possibility of weight loss not only after the storage from artificial saliva, but also in open air. Ga or Ge leakages were also detected. In addition, gallium has been shown a galvanic interaction with Ti [103] i.e. corrosion happens. Therefore, the utilization of these two alloys might not be viable at this moment.

## Biological compatibility of binary Ti-alloys

**Figure 2.**
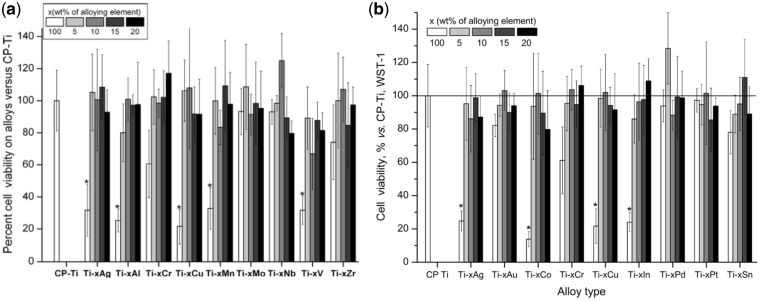
Mean ± SD cell viability for bulk pure metals and their ti based alloys versus cp-Ti after cell culture. **(a)** alloy elements: Al, Ag, V, Mn, Cr, Zr, Nb, Mo adapted from [107]; **(b)** alloy elements: Cu, in, Ag, Cr, Sn, Au, Pd, Pt, adapted from [108]

Titanium and its alloys has the properties of attracting cells, including osteoblasts and bacteria, due to various properties such as surface charge [104], existence of oxide and hydroxide groups [105], and the radicals [106]. Park *et al.* and Song *et al.* [107, 108] evaluated the biocompatibility of various alloying elements, as well as the binary Ti-alloys. The studies revealed that the cytocompatibility of pure metals ranked in the order of: Al > Ag > V > Mn > Cr > Zr > Nb > Mo > cp-Ti (Fig. 2a) and Cu > In > Ag > Cr > Sn > Au > Pd > Pt > cp-Ti (Fig. 2b). All the tested binary Ti-alloys from 5-20 wt% of alloy elements except Ti-10V have statistically similar biocompatibility with cp-Ti. On the other hand, one of the challenges for dental Ti implant is biofilm. Various techniques could be utilized such as coatings [109] and biomimetic nano-structures [110] could be applied on Ti. Indeed, in terms of Ti alloys, Ti–Ag [111] and Ti–6Al–7Nb [112] have been demonstrated certain degree of biofilm. Therefore, alloying with Ti might be beneficial to make a better material with superior mechanical and biological performance.

## Conclusion

Binary Ti alloys, in particular to Ti––Zr, Ti–In, Ti–Ag, Ti–Cu, Ti–Au, Ti–Pd, Ti–Nb, Ti–Mn, Ti–Cr, Ti–Mo, Ti–Sn and Ti–Co, with the alloying components <20% has a high potential as implant materials due to good mechanical performance without compromising the biological behaviour compare to cp-Ti. Further investigation on the inherent anti-biofilm properties could be a future topic for these alloys.

*Conflict of interest statement*. None declared.
